# Nd:Yag Laser Transluminal Embolysis: A Therapeutic Approach in Retinal Artery Occlusion

**DOI:** 10.3390/jcm13247828

**Published:** 2024-12-22

**Authors:** Bogdana Tăbăcaru, George Abboud, Mihnea Munteanu, Simona Stanca, Horia Tudor Stanca

**Affiliations:** 1 Department of Ophthalmology, “Carol Davila” University of Medicine and Pharmacy, 050474 Bucharest, Romania; bogdana.tabacaru@umfcd.ro (B.T.); horia.stanca@umfcd.ro (H.T.S.); 2 Clinical Department of Ophthalmology, “Prof. Dr. Agrippa Ionescu” Emergency Hospital, 011356 Bucharest, Romania; george.abboud999@gmail.com; 3 Clinical Department of Ophthalmology, Arad Regional Clinical Emergency Hospital, 310037 Arad, Romania; 4 Clinical Department of Ophthalmology, “Victor Babes” University of Medicine and Pharmacy, 300041 Timisoara, Romania; mihnea.munteanu@umft.ro; 5 Clinical Department of Pediatry, “Carol Davila” University of Medicine and Pharmacy, 050474 Bucharest, Romania

**Keywords:** branch retinal artery occlusion, central retinal artery occlusion, intraluminal embolysis, transluminal Nd:YAG laser embolysis

## Abstract

**Background**: Central and branch retinal artery occlusion (CRAO and BRAO) are critical causes of acute vision loss, predominantly affecting older adults with systemic vascular pathology. These occlusions typically result from embolic events, leading to partial or complete retinal ischemia. **Methods**: This retrospective case series report details of our 10-year experience using the 1064 nm Nd:YAG laser for Transluminal Nd:YAG Embolysis (TYE) in order to lyse visible emboli within the retinal arteries. **Results**: We conducted a retrospective case series involving 18 patients treated by TYE for different retinal arteries occlusions between 2014 and 2024. TYE effectively restored blood flow in the occluded arteries, with variable but generally favorable visual outcomes. Our article highlights significant clinical and interventional aspects of three treated patients: a BRAO case with multiple transluminal emboli, a case of double BRAO and a CRAO case. We also report the successful use of TYE for intraluminal lysing of an embolus located outside the optic disc. The procedures were well-tolerated, with minor complications such as preretinal or vitreous hemorrhages resolving spontaneously. **Conclusions**: TYE is a minimally invasive therapeutic option for retinal artery occlusion with visible emboli, particularly when intervention occurs shortly after the onset of symptoms. Compared to observation, TYE can improve visual outcomes and reduce the risk of permanent vision loss, presenting a significant advancement in the management of this ophthalmic emergency. The worldwide experience with TYE is continuously increasing. Further research with larger cohorts is recommended to validate these results and refine the treatment protocol.

## 1. Introduction

Branch and central retinal artery occlusion (BRAO and CRAO) are ophthalmological emergencies, consisting of partial or complete retinal ischemia. These conditions are typically caused by thromboembolism, and most commonly occur in older adults with systemic vascular pathology: systemic hypertension, arterial atherosclerosis, diabetes mellitus or heart valve diseases [1,2,3].

Retinal artery occlusion causes retinal infarction, which leads to sudden and severe vision impairment. It is an eye stroke, caused by an acute blockage in a retinal artery due to an intraluminal embolus, thrombus or atherosclerotic plaque [2,4]. Retinal artery occlusion may precede life-threatening events like stroke or myocardial infarction.

In CRAO, patients typically present with an abrupt, painless decrease in vision to count fingers, hand movement or light perception level. BRAO leads to vision loss while preserving central vision [2].

Ophthalmological examination in CRAO includes: afferent pupillary defect, development of a cherry-red spot in the fovea and whitening of the surrounding retina. Fundus examination often reveals the “boxcarring” of the blood column in the retinal vessels, or the presence of intra-arterial emboli [2,3,5]. In BRAO there is a sectorial damage of the retina. The most frequent localization of the emboli is either the arteriolar bifurcations or the areas of vascular stenosis [6,7,8,9]. Fluorescein angiography typically shows either delayed filling or a complete absence of contrast material in the affected artery. Histopathologically, retinal arterial occlusion is characterized by intracellular edema in the internal retinal layers with loss of cells, which extends from the nerve fiber layer to the inner nuclear layer [6,8,10].

Historically to date, medical therapeutic options for occlusive retinal artery pathologies remain limited and largely ineffective [2,11].

Therapeutic options may include: trombolysis, nitrogen inhalation and lowering the intraocular pressure (IOP). The effectiveness of any therapeutical procedure regarding this pathology could be proven directly by obtaining the reflow through the obstructed vessel, and indirectly by visual function improvement. There are several reports in the literature regarding the lack of artery repermeabilization for most of them [5,11,12,13,14].

Transluminal Nd:YAG laser embolysis (TYE) is a therapeutic approach primarily indicated for branch occlusions but also for the central retinal artery with a visible embolus. The method uses the 1064 nm Nd:YAG laser to disrupt the intravascular embolus by focusing the laser on its surface [7]. Prior publications indicate that TYE is a safe procedure with manageable side effects and is superior to observation in cases of central retinal artery occlusion and its branches. However, potential complications include retinal or vitreous hemorrhage, retinal tears, choroidal neovascularization and epiretinal membrane formation [4,6,8].

However, the treatment’s effectiveness varies depending on the type of embolus—cholesterol (Hollenhorst plaque), fibrin-platelet or calcium emboli [2]. It is important to note that most authors recommend laser treatment only for the emboli located on the optic disc surface, due to the risk of retinal tears. However, Oh et al. reported successful TYE in a BRAO case with embolus outside the optic disc surface [15]. If the embolus is visible, TYE can be applied regardless of the embolus type.

Photodisruption of the emboli has been found to correlate with the rapid restoration of retinal blood flow and improvement in visual acuity (VA) and/or visual field for these patients [2,4,5]. To enhance the retinal arteries’ reperfusion and the functional outcomes, TYE may be combined with urokinase thrombolysis therapy or hyperbaric oxygen treatment [2,9].

The purpose of our report is to emphasize the possibility of artery desobstruction in certain cases using the photodisruptive effect of YAG laser, the TYE technique proving as a valuable method in regaining the flow through affected blood vessels.

## 2. Materials and Methods

This case series report complies with the guidelines for human studies and was conducted ethically in accordance with the tenets of the Declaration of Helsinki. The study was approved by the local Ethics Committee of “Prof. Dr. Agrippa Ionescu” Emergency Hospital (2-2014). Written informed consent was obtained from all the patients for the use of their medical records in writing scientific reports.

We retrospective analyzed the patients with different types of retinal artery occlusions evaluated and treated in the “Prof. Dr. Agrippa Ionescu” Emergency Hospital between May 2014–May 2024.

The inclusion criteria for TYE were: BRAO or even CRAO with visible embolus, consecutive visual function loss (central or peripheral). Exclusion criteria for TYE intervention were: BRAO in the single functional eye, VA better than 20/40, low-compliance and refuse for laser intervention. The first two conditions were chosen having in mind a judicious balance between risks and benefits, the last two of them are quite obvious. For patients treated with direct oral anticoagulants (DOACs), the procedure was performed after discontinuation of DOACs or replacing them with low molecular weight heparin, as recommended by the anesthesiologist or by the cardiologist.

As the retinal vessel occlusion is likely to precede acute, serious vascular ischemic events [16,17,18], such as stroke and myocardial infarction, a systemic evaluation was recommended in all cases.

This article presents a retrospective case series report of 18 patients who successfully underwent TYE, with restoring blood flow in the occluded artery or arteries.

Preoperative ophthalmic examination included for all patients: measurement of the VA, IOP, anterior segment slit-lamp biomicroscopy, dilated fundus examination and optical coherence tomography (OCT) (Cirrus HD-OCT, Carl Zeiss Meditec, Dublin, CA, USA). Perimetry, fundus color and red-free photography and fundus fluorescein angiography were not available for all patients. VA was measured both with Snellen and ETDRS charts and was recorded as logarithm of the minimum angle of resolution of best-corrected visual acuity (logMAR of BCVA).

All patients signed an informed consent for accepting the procedure. The interventions were performed under topical anesthesia, after pharmacological mydriasis. Topical and systemic CAI were administrated prior the laser procedure. A contact lens for macular view was used to focus the Nd:YAG 1064 nm laser on the embolus surface within the retinal arteries. The small bleeding from the blocked artery induced by the procedure was stopped applying proper pressure with the laser contact lens. All TYE interventions were performed by the same surgeon (Stanca TH).

We used the fundus Centralis Direct^®^ (Volk Optical, Mentor, OH, USA) contact lens in order to focus the laser beam on the embolus surface. This contact lens offers a retinal field of view of up to 26°, with an image magnification factor of 0.9× and a laser pulse magnification factor of 1.11×. The procedures were performed with the 1064 nm Nd:YAG laser (YC-1600, NIDEK Inc., Fremont, CA, USA). A key advantage of this laser system is its ability to focus the treatment beam perpendicularly on the center of the cornea together with the image of the slit lamp. Thus, it is possible to focus the target areas along the posterior pole, allowing for precise targeting at the retinal level [6,8]. The treatment involved delivering separate laser pulses, “shot by shot”, with a maximum of four pulses per session. The laser energy level was started at 0.6 mJ and was sequentially increased until fragmentation or passing of the embolus into the vitreous, but not higher than 2 mJ. The defocus between the target laser beam and the therapeutical one was set to “0” or “+125 µm” according with the surgeon’s visual needs during intervention. In some cases, the full treatment required several laser sessions.

The BCVA in the treated eye was evaluated at 3 months after the intervention for all patients, either in our clinic or reported by the local ophthalmologist. Patients who underwent the postoperative follow-up visit in our clinic had a complete ophthalmic examination, including OCT and fundus photography.

Conservative management was chosen for patients who did not meet the criteria for the YAG laser technique. Although non-invasive treatment is generally considered less effective for obstructive arterial retinal pathology, it is preferable to mere observation. The treatment included: antiplatelet therapy, vasodilators and systemic and local carbonic anhydrase inhibitor (CAI) agents. It is hypothesized that a reduction in IOP plays a permissive role in enhancing retinal arterial circulation, thereby facilitating the downstream mobilization of emboli within the vascular network. We also performed ocular massage in order to mobilize the embolus downstream.

## 3. Results

As retinal artery occlusion is a thromboembolic event, which frequently occurs in conjunction with life-threatening stroke and cardiovascular diseases, we addressed the patients for prompt examinations and interventions for systemic vascular. Several systemic conditions which preclude an individual towards vascular narrowing (Systemic Hypertension, Diabetes Mellitus, Cardiac failure, Coronary artery disease, hypercholesterolemia, hypertriglyceridemia, hyperhomocysteinemia, smoking and obesity) were found. Demographic data, risk factors and pathological antecedents of the cohort are summarized in Table 1.

Table 2 presents the pre-, intra- and postoperative characteristics of the treated eyes.

**Figure 1:**
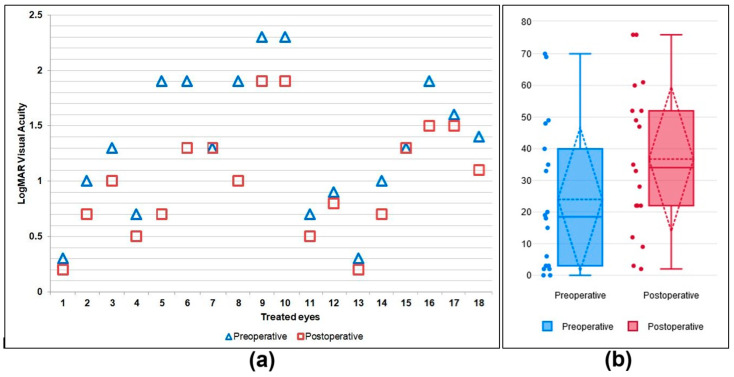
Comparison of the preoperative and the 3-month postoperative BCVA. (**a**) The logMAR visual acuities for the eighteen patients. (**b**) Box-plot of VA ETDRS score pre- and postoperation. The median ETDRS score before TYE was 18.5 letters, and the interquartile range consisted of a score of 3 letters for the first quartile and 40 for the third quartile. The minimum score was 0 letters, and the maximum was 70 letters. After the TYE intervention, the median ETDRS score was 34 letters, and the interquartile range consisted of a score of 22 letters for the first quartile and 52 letters for the third quartile. The minimum score was 2 letters, and the maximum was 76 letters.

The functional outcomes varied following TYE and were considered according to the initial BCVA (Figure 1) and to the time elapsed from the symptoms’ onset until the intervention. VA gain was best correlated with initial BCVA better than 20/200 (1 logMAR). All TYE procedures were performed after the interval of 100 min considered to be the therapeutic window. The time after symptoms onset until intervention ranged between 1 and 7 days and did not correlate with the VA gain.

Among the treated patient cohort, we selected three atypical representative cases to be detailed in order to highlight significant clinical and interventional aspects.

### 3.1. Case 1

**Figure 2:**
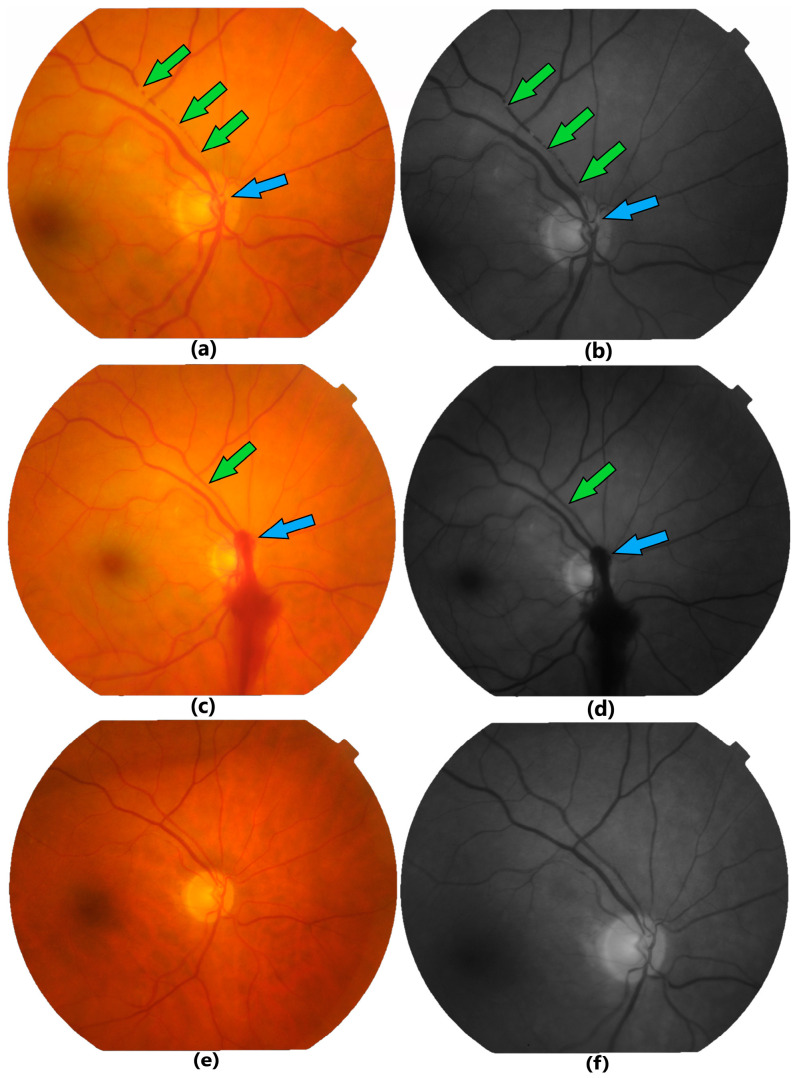
Fundus color (**a**,**c**,**e**) and red-free (**b**,**d**,**f**) photographs showing supero-temporal BRAO in case 1. (**a**,**b**) Preoperative images—one large embolus on the optic disc surface (blue arrow) and other multiple emboli along the artery (green arrows). Note the different intravascular location in the consecutive pictures (**a**,**b**) of the distal emboli indicating spontaneous mobilization. (**c**,**d**) Immediately after TYE—images showing the laser site with mild preretinal bleeding (blue arrow) and the reperfused lumen of the artery (green arrow). (**e**,**f**) The 3-month follow-up images.

A 65-year-old female presented to our clinic complaining of sudden blurry vision and inferior visual field defect in her right eye (oculus dexter, OD), symptoms that occurred 3 days before presentation. BCVA for the OD was 20/40 and for the left eye (oculus sinister, OS) was 20/20. IOP and anterior segment examination were normal in both eyes. Mydriatic fundus examination revealed OD retinal paleness in the upper region of the macula and multiple visible transluminal emboli that were located at the optic disc and along the first half of superior temporal arterial branch (Figure 2a,b). For the OS there were no pathological findings at fundus examination, except pathological arterio-venous crossings.

Successfully TYE was performed in the OD, observing intraoperative intra- and extravascular mobilization of the embolus, resulting in the restoration of blood flow in the previously occluded areas (Figure 2c,d). The small juxtapapillary preretinal hemorrhage induced by the TYE resorbed until the 3-month postoperative follow-up visit (Figure 2e,f) when BCVA for the OD was 20/30.

### 3.2. Case 2

**Figure 3:**
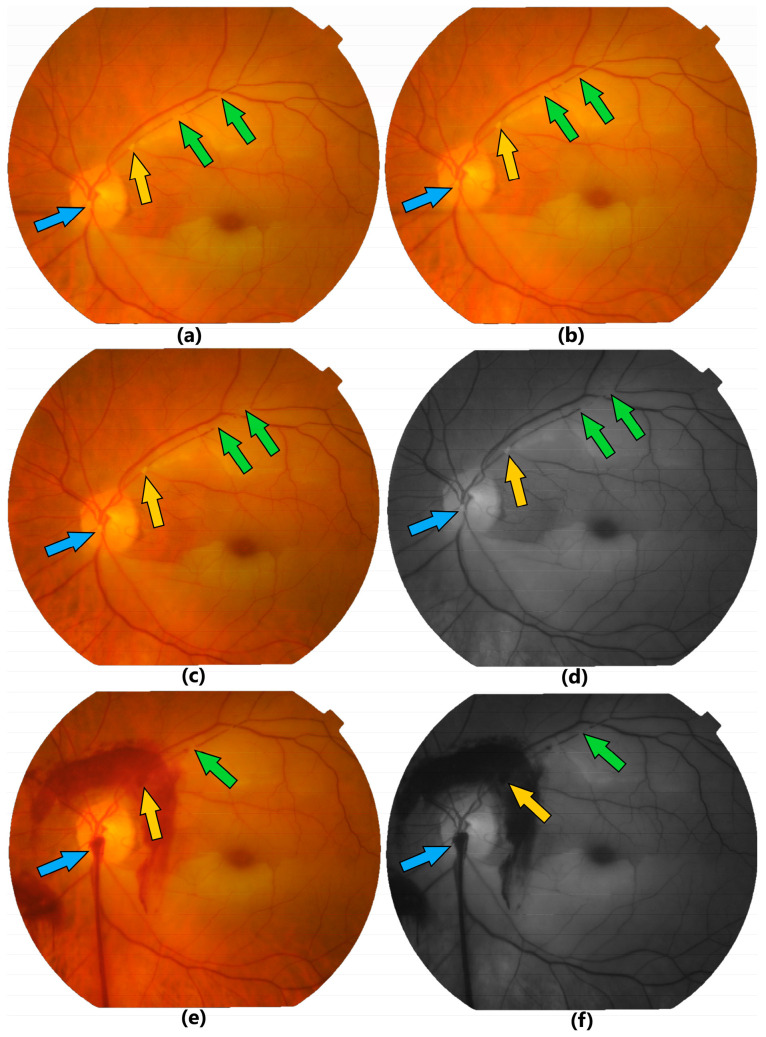
Preoperative fundus color (**a**,**b**,**c**) and red-free (**d**) photographs showing in case 2 two simultaneous BRAO of both temporal branches. One large embolus is located in the infero-temporal branch on the optic disc area (blue arrow), another large embolus is located in the supero-temporal branch at one-disc diameter outside the optic disc margin (yellow arrow) and many other smaller emboli are located downstream (green arrows). Note the different intravascular location in the consecutive pictures (**a**–**d**) of the distal emboli (green arrows) indicating spontaneous mobilization. Immediate postoperative fundus color (**e**) and red-free (**f**) photographs showing mild preretinal hemorrhages after laser action on the infero-temporal embolus (blue arrow) located on the optic disc area and on the supero-temporal first large embolus (yellow arrow) located outside the optic disc area; note the mobilized parts of lysed embolus distal to the laser shot (green arrow).

A 73-year-old female was referred from another ophthalmology clinic after being diagnosed in the OS with both superior and inferior branches retinal arteries occlusion. She was admitted in our clinic after 36 h from the embolic event. At the time of ocular examination, her BCVA was counting fingers (CF) in the OS and 20/20 in the OD. IOP and slit-lamp examinations were normal in both eyes. Dilated fundus examination demonstrated multiple emboli (Figure 3a–d) that were located at the following sites: in the inferior-temporal branch of the retinal artery—a large embolus immediately after the bifurcation from the central retinal artery; in the superior-temporal branch of the retinal artery—another large embolus approximately at one-disc diameter outside the optic disc margin and many other smaller emboli distal to the large one. Sequential fundus photographs showed spontaneous distal migration of the emboli in the superior-temporal artery (Figure 3a–d). Retinal edema was present both in inferior and superior halves of the macula, involving also the fovea. The OD had no retinal pathological findings.

The TYE procedure in the OS was first successfully completed for the embolus located near the infero-temporal arterial bifurcation, allowing complete restoration of the blood flow in this vessel. As the first laser beam was easy to be focused and allowed proper calibration of the laser energy and the correct focus of the laser beam, we decided also to treat the large embolus on the supero-temporal artery, located outside the optic disc surface. The second procedure was also successfully finished, with parts of the embolus being mobilized downstream of the vessel after one laser shot (Figure 3e,f). The patient was followed-up at the local ophthalmology clinic, with the OS BCVA after 3 months being 20/200.

### 3.3. Case 3

**Figure 4:**
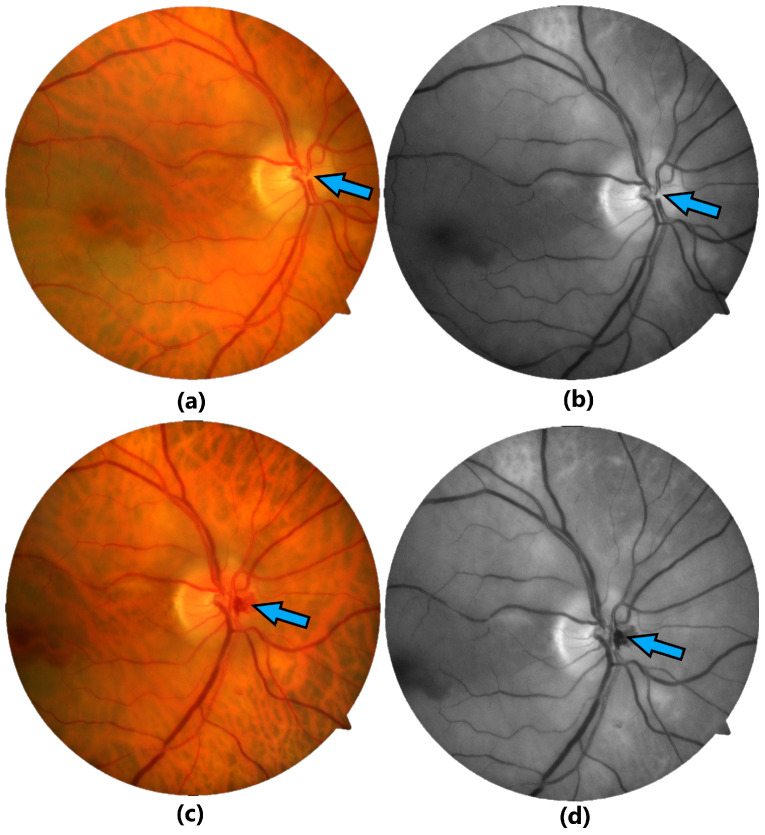
Images showing CRAO in case 3. Preoperative fundus color (**a**) and red-free (**b**) photographs showing the visible large embolus (blue arrow). Immediate postoperative fundus color (**c**) and red-free (**d**) photographs presenting the mild preretinal bleeding (blue arrow) after successful extravascular passing of the embolus into the vitreous.

A 71-year-old male presented in our emergency department complaining of a 1-day history of severe painless vision loss in the OD. BCVA was hand movement (HM) for the OD and 20/20 for the OS. IOP and anterior segment examination were normal in both eyes. Dilated fundus examination demonstrated CRAO for the OD: pale retina, cherry-red fovea and a visible large embolus at the first bifurcation (Figure 4a,b). Fundus examination in the OS showed pathological arterio-venous crossings and no other pathological findings.

The patient underwent TYE in the OD. The laser was targeted on the embolus area, at the first bifurcation of the central retinal artery. The successful laser shot flushed the embolus into the vitreous and there was an immediate bleeding at the laser site, which resolved after ocular pressure with the laser contact lens (Figure 4c,d). The patient was referred to the local ophthalmologist for the postoperative 3-month follow-up visit. Postoperative BCVA was CF.

## 4. Discussion

BRAO and CRAO are ophthalmological emergencies that lead to severe vision impairment. Non-invasive management of retinal artery occlusion includes: eyeball massage, intraocular pressure lowering medication, anterior chamber paracentesis, anticoagulation, vasodilation, thrombolysis, isovolumic hemodilution, hyperbaric oxygen treatment and tissue plasminogen activator. Unfortunately, these medical treatment options failed in proving influence on the natural history of this disorder [5,11,12,13,14].

Intraocular in situ embolectomy by pars plana vitrectomy was reported in 2020 by Hernandez-Da Mota et al. in a cilioretinal arterial branch occlusion case [19].

Several reports demonstrated that for the arterial branch occlusions with visible emboli, laser interventions can achieve reperfusion of the arterial lumen. The first laser treatment was reported in 1989 by Dutton et al. who melted cholesterol emboli with argon laser in a BRAO case, but this method failed to produce significant functional improvement [6,7,20].

Thirteen years later, in 2002, Opremcak and Benner introduced the use of a 1064 nm photodisruptive Nd:YAG laser for the selective lysis of solid intravascular emboli without damaging the vascular walls [7]. Despite limited global experience and a small number of reported procedures, this technique has proven to be an effective intervention for treating recent BRAO and CRAO caused by visible emboli [4].

The first TYE procedure reported in Romania and Europe was performed in 2006, by Stanca TH. This procedure successfully treated an inferior BRAO [21]. Since then, many cases of retinal arteries occlusion have been treated by the same surgeon using the TYE technique. This article summarizes the TYE procedures performed by Stanca TH between 2014 and 2024. It is the second case series report in Romania after the one published by the same author in 2014, which analyzes the previously treated cases between 2006 and 2014 [6]. To the best of our knowledge, Stanca TH is the first and only surgeon in Romania who reported TYE procedures.

The functional outcome varied following TYE, VA gain being correlated to initial BCVA better than 20/200 (1 logMAR).

Among the eighteen cases that underwent TYE procedure, we detailed three atypical representative cases in order to highlight significant clinical and interventional aspects.

The first case demonstrates a BRAO with multiple transluminal emboli located along the supero-temporal branch from the optic disc until the half of the branch. Therapeutic laser shot was applied only on the largest embolus located on the optic disc, breaking it in smaller parts that had pushed the other emboli downstream, allowing immediate reperfusion of the artery.

The second patient is a rare case with double BRAO. The infero-temporal branch was occluded by a large embolus in the optic disc area. The supero-temporal branch had multiple intraluminal visible emboli, the largest approximately at a one-disc diameter outside the optic disc margin and many other smaller emboli downstream. The TYE procedure was first performed on infero-temporal embolus, located on the optic disc. The laser shots used to break this embolus allowed the correct focus of the laser beam and proper calibration of the energy, so we decided also to treat the large embolus on the supero-temporal branch, outside the optic disc surface. The second procedure was also successfully performed, without complications, except mild bleeding at the site of laser action. Both emboli were broken intravascularly and the fragments were mobilized downstream the vessels.

The third case presents the TYE procedure for a CRAO with a visible large embolus. The successful laser shot flushed the embolus into the vitreous.

Preretinal or mild vitreous hemorrhages were observed in almost all cases. Bleeding was resolved by ocular pressure with the laser contact lens immediately after completing the procedure. No other complication occurred.

The timing of embolus lysis is crucial and should be performed as soon as possible after the onset of the occlusion to maximize the likelihood of regaining visual function [1,5]. Hayreh et al. demonstrated that retina can tolerate acute ischaemia up to 98 min with full recovery [22]. Ischemic changes in the retina becomes irreversible 100 min after the embolic event [1,5,22]. Unfortunately, most patients are referred after the therapeutic window with at least 1–2 days. However, as the obstruction may be incomplete, some retinal areas may have reversible injury or may remain viable, allowing for potential recovery if the artery is reperfused [5]. Furthermore, the retina may longer tolerate ischemia in patients with residual retinal circulation [22], for instance when a cilioretinal artery is present. Therefore, similar to our reported cases, many authors performed laser interventions even after 7 days after the occlusive event [2,8,9,11].

The major limitation of our study is the small sample size of 18 cases, which is insufficient for reliable inferential statistics and statistical significance testing. Thereby, this article presents our results as a case-series report.

## 5. Conclusions

Medical therapeutic options for occlusive retinal artery pathologies include: trombolysis, nitrogen inhalation and lowering the intraocular pressure (IOP). Unfortunately, there are several reports demonstrating that non-invasive management of retinal artery occlusion failed in proving influence on the natural history of this disorder [2,5,11,12,13,14].

TYE is a pioneering technique for retinal artery occlusion with visible embolus. From the first reported case to date, the worldwide experience with TYE is continuously increasing. To the best of our knowledge, after more than 20 years from the first procedure in 2002, there are about one hundred reported cases of TYE worldwide [2,3,5,8,9,11,15,23].

As the visual prognosis is low in the absence of an effective conventional treatment, TYE may be beneficial in selected cases of retinal artery occlusion. The proper application of the TYE procedure breaks up the embolus, enhancing the restoration of blood flow in the retinal arteries. Nd:YAG laser embolysis may be a safe and effective method in BRAO and CRAO cases with visible emboli. The procedure definitely has risks and complications but if performed correctly, it is superior to observation.

Random trials for precisely identifying the risk-benefit ratio of TYE in the treatment of retinal occlusion pathology will be welcomed, but there is an ethical aspect (treated versus no treated patients), which should be properly analyzed.

## Figures and Tables

**Table 1:** Summary of the demographic, risk factors and pathological antecedents for the eighteen patients.

Parameter	Results
Gender	Female (*n* = 7, 38.89%) Male (*n* = 11, 61.11%)
Age	Mean age = 76.2 years Age range = 65–87 years
Risk factors	Hypercholesterolemia (*n* = 16, 88.89%) Hypertriglyceridemia (*n* = 15, 83.33%) Hyperhomocysteinemia (*n* = 15, 83.33%) Smoker/Former smoker (*n* = 14, 77.78%)
Pathological antecedents	Systemic hypertension (*n* = 18, 100%) Diabetes Mellitus (*n* = 12, 66.67%) Cardiac failure (*n* = 11, 61.11%) Depression (*n* = 9, 50%) Coronary artery disease (*n* = 8, 44.44%) Benign prostatic hyperplasia (*n* = 8, 44.44%) Chronic kidney disease (*n* = 7, 38.89%) Atrial fibrillation (*n* = 6, 33.33%) Obesity (*n* = 5, 27.78%) Glaucoma (*n* = 4, 22.22%) Chronic obstructive pulmonary disease (*n* = 4, 22.22%) Hypothyroidism (*n* = 4, 22.22%) Osteoporosis (*n* = 4, 22.22%) Oncologic pathology (*n* = 3, 16.67%) Cirrhosis (*n* = 3, 16.67%) Gout (*n* = 3, 16.67%) Chron’s disease (*n* = 2, 11.11%) Psoriasis (*n* = 1, 5.55%)

**Table 2:** Summary of the ophthalmological characteristics for the treated eyes.

Parameter	Results
Affected eye	OD (*n* = 10, 55.56%) OS (*n* = 8, 44.44%)
Occlusion site/sites	Superotemporal BRAO (*n* = 9, 50%) Inferotemporal BRAO (*n* = 6, 33.33%) Superotemporal + Inferotemporal BRAO (*n* = 1, 5.55%) Hemiretinal artery (*n* = 1, 5.55%) CRAO (*n* = 1, 5.55%)
Location of the embolus/emboli	On the optic disc (*n* = 17, 94.45%) On the optic disc and outside the optic disc (*n* = 1, 5.55%)
Probable embolus type	Fibrinoplatelet (*n* = 6, 33.33%) Cholesterol (*n* = 5, 27.78%) Calcium (*n* = 4, 22.22%) Cholesterol and fibrinoplatelet (*n* = 3, 16.67%)
Time of TYE after occlusion onset	1 day (*n* = 2, 11.11%) 2 days (*n* = 10, 55.55%) 3–4 days (*n* = 3, 16.67%) 5–6 days (*n* = 2, 11.11%) 7–8 days (*n* = 1, 5.55%)
Type of embolus dislodge	Extravascular (*n* = 9, 50%) Intravascular and extravascular (*n* = 5, 27.78%) Intravascular (*n* = 4, 22.22%)
Complications	Preretinal with mild vitreous hemorrhage (*n* = 18, 100%)

Abbreviations: OD—oculus dexter, OS—oculus sinister, BRAO—branch retinal artery occlusion, CRAO—retinal artery occlusion.

## Data Availability

The raw data supporting the conclusions of this article will be made available by the authors on request.
